# Survival following lobectomy *vs* limited resection for stage I lung cancer: a meta-analysis

**DOI:** 10.1038/sj.bjc.6602414

**Published:** 2005-03-08

**Authors:** H Nakamura, N Kawasaki, M Taguchi, K Kabasawa

**Affiliations:** 1 Department of Chest Surgery, Atami Hospital, International University of Health and Welfare, 13-1 Higashikaigan-cho, Atami-shi, Shizuoka, 413-0012, Japan; 2 Center for Medical Informatics, International University of Health and Welfare, 2600-1 Kitakanamaru, Ohtawara-shi, Tochigi, 324-8501, Japan

**Keywords:** lung cancer, limited resection, segmentectomy, wedge resection, meta-analysis

## Abstract

Extent of resection needed to treat lung cancer has long been an issue. The sole randomised controlled trial, reported by the Lung Cancer Study Group, advised against limited resection as standard surgery even for small peripheral non-small-cell lung cancers (⩽3 cm), because of frequent local recurrences. Elsewhere, conflicting results have been reported from different institutions. We therefore conducted a meta-analysis of reported studies to compare survival of stage I patients between limited resection and standard lobectomy. A MEDLINE web search for computer-archived bibliographic data yielded 14 articles suitable for analysis. Combined survival differences (survival rate with lobectomy minus that with limited resection) at 1, 3, and 5 years after resection according to the DerSimonian–Laird random effects model were 0.7% (95% CI, −0.8 to 2.1; *P*=0.3659), 1.9% (95% CI, −3.7 to 7.4; *P*=0.5088), and 3.6% (95% CI, −0.4 to 10.5; *P*=0.3603), respectively. None of these survival differences were significant, indicating that survival after limited resection for stage I lung cancer was comparable to that after lobectomy. However, since interstudy heterogeneity was detected, caution is required in interpretation of the results.

Limited resection for lung cancer was proposed in the early 1970s (Le Roux, 1972). Acceptable results of segmentectomy in a large number of patients were reported by Jensik *et al* (1973). Since then, several retrospective studies (Breyer and Jensik, 1985; Stair *et al*, 1985; Jensik, 1987; Temeck *et al*, 1992) considering efficacy of limited resection have been reported. In 1995, the Lung Cancer Study Group (LCSG) published final results of a randomised controlled trial (RCT) (Ginsberg and Rubinstein, 1995; Lederle, 1996) comparing local recurrence and survival after limited resection with those after standard lobectomy for stage IA non-small-cell lung cancer (NSCLC). Representing the only randomised trial worldwide to address the question of whether limited resection truly is comparable to standard lobectomy, that trial showed frequent locoregional recurrences and a tendency toward poorer survival in the limited resection group.

Although the randomised trial (Ginsberg and Rubinstein, 1995) concluded that limited resection should not be standard surgery even for small peripheral NSCLC, several surgeons (Kodama *et al*, 1997; Tsubota *et al*, 1998; Koike *et al*, 2003) have continued to perform some intentional limited resections. Indeed, results reported from various institutions up to now have been conflicting. Considering recent implementation of minimally invasive surgical techniques such as video-assisted thoracoscopic surgery (VATS), efficacy of limited resection in small, node-negative NSCLC needs to be re-evaluated (Sugarbaker and Strauss, 2000).

We therefore conducted a meta-analysis of published studies to quantitatively review survival data for limited resection of lung cancer in comparison with data for standard lobectomy.

## MATERIALS AND METHODS

### Eligibility criteria for meta-analysis

This meta-analysis was limited to studies comparing survival data of limited resection with those of standard lobectomy. The following eligibility criteria were established before collecting articles: (1) ‘Limited resection’ was defined as sublobular resection, including wedge resection and segmentectomy. (2) Operative approaches could include either thoracotomy or VATS. (3) Survival rates for a specific time interval after operation were stated in the article. (4) Study subjects had to be limited to clinical stage I patients. (5) Median follow-up time was to exceed 2 years. (6) Articles were published in English in the periodical medical literature from 1970 to August 2004. (7) When multiple articles by the same author or study group analysed the same series of patients, a single most informative article was chosen for the meta-analysis.

### Collection of published studies

The MEDLINE web search for computer-archived bibliographic data concerning limited resection and postoperative survival in lung cancer was primarily performed in August 2004. Keywords lung ‘cancer+limited resection’, ‘lung cancer+wedge resection’, ‘lung cancer+segmentectomy’, and ‘limited resection +lobectomy’ hit 627, 344, 193, and 117 citations, respectively. Manual selection of relevant studies was carried out based on the summary analysis. Overlapping or unrelated articles were excluded, and items from hand-searched bibliographies were added. Of 18 articles initially found by the methods above, two were excluded for being reported by the same author or study group analysing a series of patients more informatively considered in another article. In three articles, advanced disease stages were included. One report of these three also was among the two representing overlap. Thus, four articles (Errett *et al*, 1985; Pastorino *et al*, 1991; Sugarbaker and Strauss, 2000; Miller *et al*, 2002) were excluded (Table 1), while 14 articles fulfilled eligibility criteria.

### Statistical analyses

DerSimonian–Laird random effects analysis (DerSimonian and Laird, 1986) was used to estimate the survival difference (i.e. survival rate after standard lobectomy minus that of limited resection) at the end points of 1, 3, and 5 years after operation. Generally used to combine heterogeneous studies, this method produces a combined survival difference and a 95% confidence interval with a heterogeneity test at each end point. Survival rates were derived from published survival curves when not provided explicitly in the text or tables. Subjects censored prior to each end point were subtracted from the denominators (number of patients for follow-up), giving a conservative confidence interval for the summary statistic. Censored cases were counted by placing tick marks on survival curves when provided, as described by Mitsudomi *et al* (2000). The correlation coefficient (*r*) was calculated to examine the relationship between two variables. Significance was tested by the Bartlett test. For these tests, a *P*-value <0.05 was considered significant.

Publication bias was tested by the method of Egger *et al* (1997); for this, a *P*-value <0.1 was considered significant.

## RESULTS

A total of 14 studies (Hoffmann and Ransdell, 1980; Read *et al*, 1990; Date *et al*, 1994; Warren and Faber, 1994; Ginsberg and Rubinstein, 1995; Harpole *et al*, 1995; Lederle, 1996; Kodama *et al*, 1997; Landreneau *et al*. 1997; Pastorino *et al*, 1997; Kwiatkowski *et al*, 1998; Okada *et al*, 2001; Koike *et al*, 2003; Campione *et al*, 2004; Keenan *et al*, 2004) served as data sources for the present meta-analysis (Table 2). Their designs were retrospective in 12, matched-pair in one, and RCT in one. Limited resection was performed for a total of 903 patients, while comparable standard lobectomy was performed for 1887 patients. Overall classification of histologic types including additional 125 pneumonectomies in three studies (Harpole *et al*, 1995; Pastorino *et al*, 1997; Kwiatkowski *et al*, 1998) were 878 squamous cell carcinomas and 1617 nonsquamous cell carcinomas. Histologic types were not mentioned in two studies (Landreneau *et al*, 1997; Keenan *et al*, 2004) including 420 patients. Stages and tumour–nodes–metastasis (TNM) profiles of patients who underwent limited resection were IA (T1N0M0) and IB (T2N0M0).

**Figure 1:**
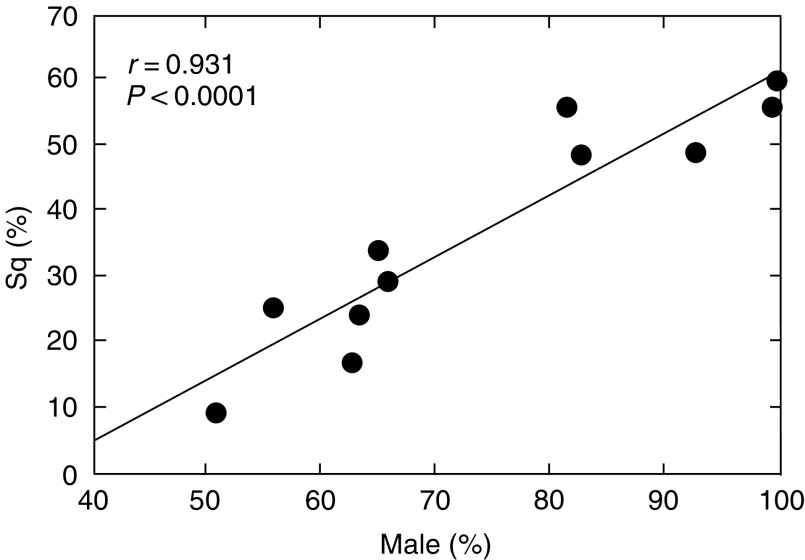
A strong correlation is evident between percentages of squamous cell carcinoma in the various studies and those of male patients (*r*=0.931, *P*<0.0001).

Studies included were considered highly heterogeneous for the following reasons. The percentage of nonsquamous cell carcinoma in each study ranged from 39.7% (Hoffmann and Ransdell, 1980) to 90.5% (Koike *et al*, 2003). Further, the percentage of male patients in each study ranged from 50.6% (Koike *et al*, 2003) to 100% (Hoffmann and Ransdell, 1980). Percentages of squamous cell carcinoma in each study showed strong association with male gender (Figure 1, *r*=0.931, *P*<0.000l). The reason for limited resection differed from study to study; the most frequent reason was poor cardiopulmonary function in seven studies; limited resection was intentional in four studies and was part of an RCT design in one. The reason was not clearly mentioned in 3 studies. All of these differences might affect the respective studies and contribute to interstudy heterogeneity in the present meta-analysis.

**Figure 2:**
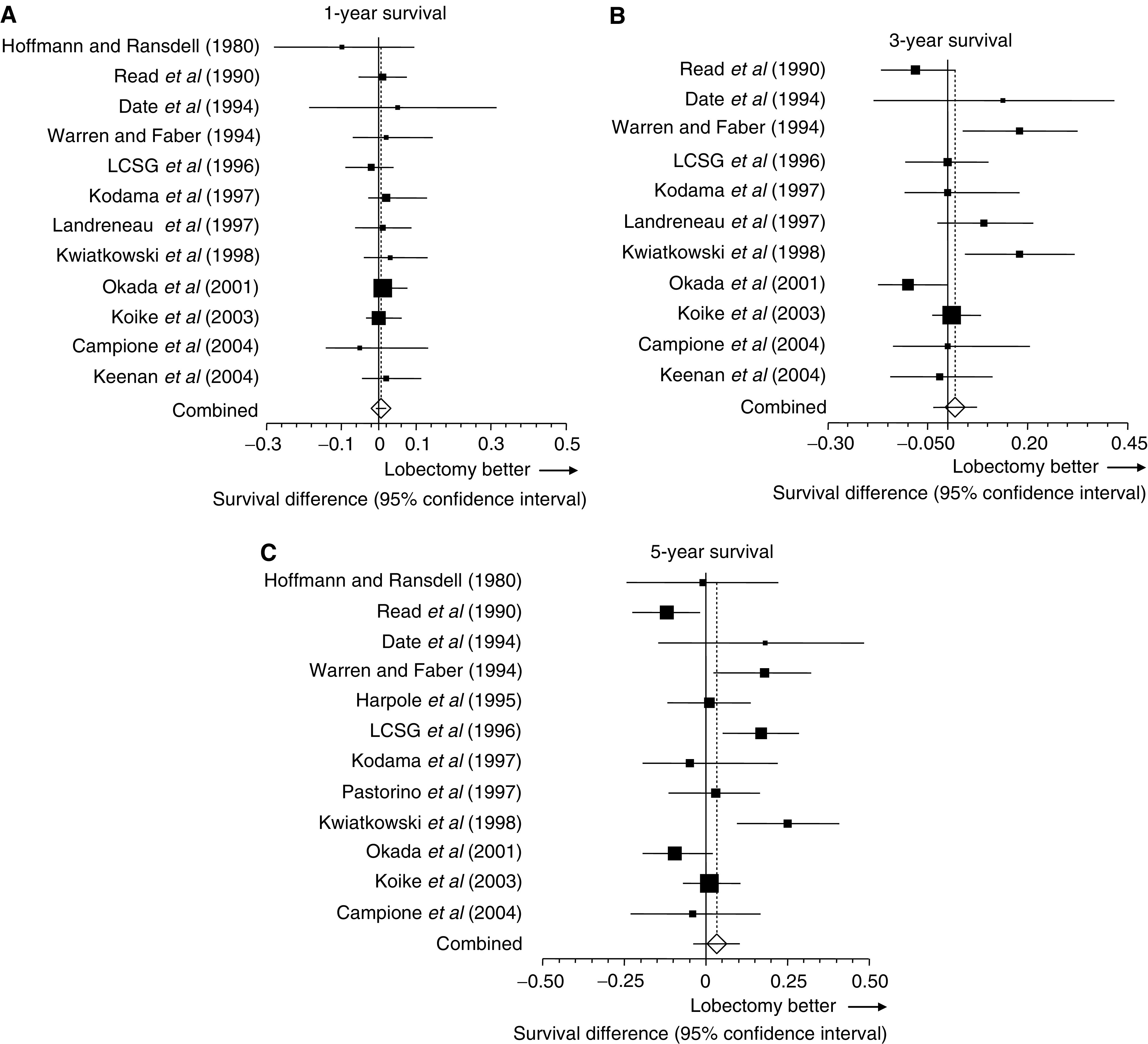
Meta-analysis of survival differences between limited resection and lobectomy. Bars, 95% CI of survival rates in patients with lobectomy minus those in patients with limited resection. Areas of squares are proportional to weights used for combining data. The centre of the lozenge gives the combined survival difference. The survival difference was considered statistically significant if the 95% CI for the overall survival difference did not overlap zero. (**A**) The combined survival difference at 1 year was 0.7% (95% CI, −0.8 to 2.1; *P*=0.3659). The 1-year survival rate was not available in studies by Harpole (1995) and Pastorino (1997). (**B**) The combined survival difference at 3 years was 1.9% (95% CI, −3.7 to 7.4; *P*=0.5088). The 3-year survival rate was not available in studies by Hoffmann (1980), Harpole (1995), and Pastorino (1997). (**C**) The combined survival difference at 5 years was 3.6% (95% CI, −0.4 to 10.5; *P*=0.3603). The 5-year survival rate was not available in studies by Landreneau (1997) and Keenan (2004). *Q*-statistics and *P*-value for the heterogeneity test at 1, 3, and 5 years were as follows: (*Q*=4.6, *P*=0.9471; *Q*=27.0, *P*=0.0026; and *Q*=33.6, *P*=0.0004).

Combined survival differences at 1, 3, and 5 years after resection were 0.7, 1.9, and 3.6%, respectively (Figure 2A–C). None of these combined survival differences were significant (see legend to Figure 2). Heterogeneity testing indicated that studies were heterogeneous at 3- and 5-year time points (see legend to Figure 2).

Publication bias was not detected at 1, 3, or 5 years; all *P*-values >0.1; 0.5402, 0.1807, and 0.3633, respectively.

## DISCUSSION

The extent of lung resection most appropriate for small cancers has been discussed for a number of decades. Although limited resection for patients with poor cardiopulmonary reserve is regarded reasonable, intentional limited resection for patients expected to withstand standard lobectomy has not been established. We therefore performed a meta-analysis to examine published data. Some authors (Stewart and Parmar, 1993) are considering meta-analysis based on individual patient's data is the best. However, we did not take that approach because collecting all those data is hard to accomplish, and needs exhaustive labour.

As meta-analysis originally was developed to combine the results of RCTs (Yusuf *et al*, 1985), applying this methodology to suitability of limited resection for lung cancer is problematic. Since we could find only one RCT, we included additional 13 retrospective studies to obtain the summary statistics. Consequently, we found significant variation between studies for tumour size, distribution of histologic types, male/female ratio, reasons for limited resection, and details of the operation. All of these differences might contribute to interstudy heterogeneity, and indeed the heterogeneity test detected considerable heterogeneity between combined studies at time points 3 and 5 years after resection.

Publication bias (Egger *et al*, 1997) also is a problem in meta-analysis, but this was not detected in our present study; the selected articles apparently were reasonably representative of the actual average.

Combining 14 published reports, we concluded that while survival after lobectomy was slightly better than that after limited resection at 1, 3, and 5 years postoperatively, the differences were not statistically significant. Since most included articles were retrospective studies, we should interpret the present results carefully.

We believe that the most important factor affecting results in each study was the choice of indications for limited resection. In this meta-analysis, the most frequent reason was poor cardiopulmonary reserve. If so, overall survival in limited resection should be worse than that in lobectomy, since many poor-risk patients will die of diseases other than lung cancer. In a single-institution study, overall 5-year survival in patients undergoing limited resection because of such compromise (48%) was significantly lower than that of patients undergoing intentional limited resection (93%) (Kodama *et al*, 1997). However, if prognosis is evaluated by cancer-specific survival (CSS), the survival rate should be higher in compromised patients because expected deaths from lung cancer might be diminished by prior deaths from cardiopulmonary diseases. In the present study, since two studies (Read *et al*, 1990; Harpole *et al*, 1995) evaluated CSS after operation, good prognosis after limited resection might have been overevaluated. In most studies, limited resection was performed for smaller nodules than those resected by lobectomy, which in itself would favour better survival in the limited resection group, since the postoperative prognosis with IA disease is better than with IB. Specifications of operative procedures also are important. In intentional segmentectomy studies (Okada *et al*, 2001; Koike *et al*, 2003), nodal metastases were carefully sought during the operation, and cases with these were strictly excluded from limited resection. This procedure would yield a different limited resection group from that obtained by simple wedge resection without intraoperative nodal examination. Of 89 patients (tumour ⩽2 cm) scheduled for limited resection in an intentional study (Okada *et al*, 2001), 19 patients (21.3%) had to undergo different procedures because nodal involvement was found in 12 and local invasion was found in seven. A relatively high frequency of lymph node metastases from small lung cancers strongly suggests that tumour size alone is not a good criterion for limited resection.

Histologic type of the tumour also may affect results of limited resection. In our study (Nakamura *et al*, 2004), analyzsng 100 patients who underwent limited resection without systematic lymph node dissection, the overall 5-year survival rate for 73 patients with small adenocarcinomas (⩽2 cm) was 93.7%, which was significantly better than for those with larger adenocarcinomas (24.8%). In addition, we found the overall 5-year survival rate for patients with well-differentiated adenocarcinoma (81.2%) to be significantly better than for a group combining moderately and poorly differentiated adenocarcinomas (30.7%). Thus, in addition to tumour size, biologic characteristics importantly affect survival after limited resection. Since the most reliable results are likely to be those of the RCT, we believe that limited resection truly is inferior to lobectomy in terms of locoregional recurrence and survival in a study where eligibility for randomisation depends solely on tumour size (⩽3 or ⩽2 cm). However, we also would maintain that a subset of NSCLC can be resected completely by limited resection. One example would be a small, slowly growing, localised bronchioloalveolar carcinoma showing only ground-glass opacity (GGO) on computed tomography (Kaneko *et al*, 1996; Sone *et al*, 1998). In our opinion, these lesions can be resected completely by VATS wedge resection (Watanabe *et al*, 2002; Nakamura *et al*, 2004), given their low invasiveness and absence of lymph node metastases (Nakata *et al*, 2002).

In conclusion, the present meta-analysis of published data disclosed that survival after limited resection for stage I lung cancer is comparable to lobectomy. However, considerable heterogeneity among studies suggests that clinicopathologic features of patients who underwent limited resection in the studies analysed were quite different. We believe that some lung cancers can be cured by limited resection, if we can identify tumours of minimally invasive nature, such as small bronchioloalveolar carcinomas diagnosed by CT (Nakamura *et al*, 2004). Further clinicopathologic studies of the biologic nature of various lung cancers should help to address this problem.

## Figures and Tables

**Table 1:** Studies excluded from the present meta-analysis

**Authors**	**Study design**	**Stage**	**No. of limited resection**	**No. of lobectomy**	**Reasons for exclusion**	**Survival difference**
Errett *et al* (1985)	RS	IA+B				
		IIA	100 (W)	97	Included advanced and unknown stages	NS
		Unknown				
Pastorino *et al* (1991)	RS	IA+B	61 (S+W)	411	Up dated by Pastorino *et al* (1997)	NS
Sugarbaker and Strausss (2000)	Review	IA+B	58 (S+W)	172	Same series of patients was reported by Kwiatkowski *et al* (1998)	Lobectomy better
Miller *et al* (2002)	RS	IA+IB				
		IIA			Included advanced stages	Lobectomy better
		IIIA	25 (S+W)	75		
		⩽1 cm				

RS=retrospective study; S=segmentectomy; W=wedge resection; ND=not described; NS=not significant.

**Table 2:** Studies included in the present meta-analysis

**Authors**	**Study design**	**Stage**	**No. of limited resection**	**No. of lobectomy**	**Reasons for limited resection**	**Survival difference**
Hoffmann and Ransdell (1980)	RS	IA	33 (W)	40^a^	Poor cardiopulmonary function and smaller lesions	NS
Read *et al* (1990)	RS	IA	113 (1O7S+6W)	131	ND	NS (CSS)
Date *et al* (1994)	MPS	IA	16 (6S+10W)	16	Poor pulmonary function	Lobectomy better
Warren and Faber (1994)	RS	IA+B	66 (S)	103	Poor cardiopulmonary function and smaller lesions	Lobectomy better
Harpole *et al* (1995)	RS	IA+B	75 (W)	193	Poor cardiopulmonary function and smaller lesions	NS (CSS)
LCSG (1996)	RCT	IA	122 (82S+40W)	125	Randomisation	NS
Kodama *et al* (1997)	RS	IA	46^b^ (W)	77	Intentional resection for small lesions	NS
Landreneau *et al* (1997)	RS	IA	102 (W)	117	Poor cardiopulmonary function	NS
Pastorino *et al* (1997)	RS	IA+B	53 (S+W)	367	ND	NS
Kwiatkowski *et al* (1998)	RS	IA+B	58 (S+W)	186^c^	ND	Lobectomy better
Okada *et al* (2001)	RS	IA⩽2 cm	70 (S)	139	Intentional resection for small lesions ⩽2 cm	NS
Koike *et al* (2003)	RS	IA⩽2 cm	74 (60S+14W)	159	Intentional resection for small lesions ⩽2 cm	NS
Campione *et al* (2004)	RS	IA	21 (S)	100	Poor cardiopulmonary function	NS
Keenan *et al* (2004)	RS	IA+B	54 (8)	147	Poor pulmonary function	NS

a Tumours peripherally located.

b Only intentional resection.

c Including 13 pneumonectomies.

LCSG=Lung Cancer Study Group; S=segmentectomy; W=wedge resection; ND=not described; NS=not significant; MPS=matched-pair study; RCT=randomised controlled trial; RS=retrospective study; CSS=cancer-specific survival.
